# A Longitudinal Study of Stress, Alcohol, and Blood Pressure in Community-Based Samples of Blacks and Non-Blacks

**Published:** 1999

**Authors:** Marcia Russell, M. Lynne Cooper, Michael R. Frone, Robert S. Peirce

**Affiliations:** Marcia Russell, Ph.D., is a senior research scientist at the Prevention Research Center, Berkeley, California. M. Lynne Cooper, Ph.D., is an associate professor in the Department of Psychology, University of Missouri at Columbia, Columbia, Missouri. Michael R. Frone, Ph.D., is a senior research scientist and Robert S. Peirce, Ph.D., is a research scientist, both at the Research Institute on Addictions, State University of New York at Buffalo, Buffalo, New York

**Keywords:** African American, blood pressure, AOD (alcohol or other drug) use, psychological stress, New York, hypertensive disorder, emotional and psychiatric depression, anxiety state, coping skills, racial differences, gender differences, literature review, longitudinal study, survey

## Abstract

Both alcohol use and stress appear to increase blood pressure. In addition, stress is associated with increased alcohol use. To investigate these relationships, researchers interviewed representative samples of the black and non-black adult household populations in Erie County, New York, in 1986, 1989, and 1993. The results support a causal relationship between stress and alcohol use and point to a number of factors that influence this relationship. Significant relationships between changes in alcohol use and blood pressure were also found. Although the researchers found little evidence for a direct effect of stress on blood pressure, stress related to family life, anxiety, and depression was associated with an increased risk for hypertension.

Epidemiologic studies strongly support the hypothesis that regular alcohol use above a poorly defined threshold is related to elevated blood pressure (Klatsky 1995). Other theories suggest that psychological stress affects hormone levels that are associated with elevated blood pressure (Henry et al. 1995). However, researchers believe that people increase their alcohol use to relieve tension or depression caused by exposure to stress and to things that cause stress (i.e., stres-sors). Thus, a key question to consider is the extent to which high blood pressure associated with psychological stress can be explained by increased alcohol use. Because the prevalence of hypertension is higher among blacks than among other ethnic groups, we were especially interested in determining whether racial differences influence the effects of stress and alcohol use on blood pressure.

Few longitudinal studies have examined factors influencing blood pressure among blacks, and no longitudinal studies have examined the joint effects of stress and alcohol use on blood pressure. Longitudinal studies, which follow participants over time to determine the sequence of events under investigation, are critical to establishing causal relationships between risk factors and outcomes.

This article summarizes findings from a longitudinal study funded by the National Institute on Alcohol Abuse and Alcoholism (NIAAA) entitled Stress, Race, and Alcohol Use in a Household Population. The study was designed to examine the influence of stress and alcohol use on blood pressure and alcohol problems and to investigate racial differences in those relationships. The initial survey in 1986 was followed up in 1989 and 1993. Using data collected during the 7-year period enabled us to investigate the causal nature of the processes in question over time (i.e., longitudinally). The three surveys are referred to in this article as time 1, time 2, and time 3.

The study was based on a model (see figure, p. 300) of the effects of stress on alcohol use and the effects of both stress and alcohol use on alcohol abuse, alcohol dependence, and hypertension. As illustrated in the figure, the model proposed that stressors resulted in a stress response (i.e., negative feelings or a negative affect, such as depression, distress, or anxiety). The stress response appears to motivate a person to drink in an effort to cope with negative feelings, which, in turn, are exacerbated by increased alcohol use. Furthermore, sustained or habitual stress, drinking to cope, and increased alcohol use appear to cause, over an extended period of time, alcohol problems and hypertension. Because drinking tends to increase rather than relieve problems, the use of alcohol to cope with stressors is likely to increase a drinker’s exposure to stressors over time. Furthermore, as a person’s tolerance to alcohol develops, he or she drinks larger amounts of alcohol to achieve the same relief from negative feelings that he or she formerly achieved from consuming smaller alcohol doses. The inevitable result is a vicious cycle in which drinking to cope maintains and exacerbates alcohol problems.

Other factors also affect the stress process. Factors that increase the impact of a stressor on alcohol use are considered vulnerability factors, whereas factors that decrease the stressor’s impact are considered buffering factors. Vulnerability and buffering factors examined in this study include positive alcohol expectancies (i.e., the belief that drinking alcohol will help one cope with stress), intrapersonal and interpersonal resources, coping styles, and demographic characteristics. These factors may directly affect exposure to stressors, stress responses, drinking to cope, and the outcomes illustrated in the figure, or they may moderate or influence the relationships among these elements. For example, we postulated that people who rated high on positive alcohol expectancies would drink more when exposed to stress than people who rated low on positive alcohol expectancies. Thus, positive alcohol expectancies would act as a vulnerability factor that moderated the relationship between stressors and alcohol use. Several psychological theories of drinking and alcoholism contributed to the development of this model (Blane and Leonard 1987).

## Methods: Survey Sample and Response Rates

We selected samples that were representative of both the black and non-black adult (i.e., age 18 and older) household populations of Erie County, New York, using a stratified three-stage design. U.S. Census blocks were stratified according to their racial and educational composition. Within each stratum, the three stages of selection were: (1) U.S. Census blocks, (2) housing units, and (3) an adult resident within each housing unit. Households were characterized by race (i.e., black and non-black) and educational level (i.e., low, medium, and high). For non-blacks with low, medium, and high levels of education, the response rates were 81.0, 72.7, and 75.0 percent, respectively. Among blacks with low, medium, and high levels of education, the response rates were 76.9, 80.7, and 80.4 percent, respectively.

The table on p. 301 shows the race and gender of participants surveyed at times 1, 2, and 3. No significant differences existed in followup rates related to race, and no significant interactions occurred between race and gender. Women, however, were more likely than men (*p* < 0.01) to have participated in followup surveys. A poststratified sampling weight was computed to adjust parameter estimates for differential selection probabilities attributable to household size and for disproportionate sampling based on gender, race, and education.

Trained interviewers surveyed respondents in their homes and measured their body weights and heights. The interviewers also measured respondents’ blood pressure using procedures recommended by the American Heart Association (Russell et al. 1991).

## Findings

Because the stress-alcohol model was too complex to be tested in a single analysis, we tested specific relationships predicted by the model in separate analyses. These relationships are examined separately in the following sections.

### Stress and Alcohol Use

The surveys asked participants about a number of stressors, including negative life events experienced in the past year, problems experienced within the past month, financial-related stress, and stressors related to important roles that the participants maintained in their daily lives (e.g., employee, spouse, and parent). The measure of negative life events was based on 52 life events organized into 8 domains: work, love and marriage, children, finances, health and illness, criminal and legal matters, household, and school. Respondents rated the impact of events experienced in the previous 12 months on a scale ranging from 1 (extremely negative) to 6 (extremely positive). The negative life events measure was the sum of events scored as 1, 2, or 3. Having experienced negative life events in the past year was significantly and positively related to drinking to cope, to alcohol consumption in the past year, and to alcohol-related problems in the past year (Cooper et al. 1992), as were both acute financial problems and chronic financial strain (Peirce et al. 1994).

#### Moderators of the Relationship Between Stress and Alcohol Use

Some people are more likely than others to drink alcohol in response to stress, suggesting that individual factors moderate the relationship between stress and alcohol use. In this study, exposure to stressors was consistently associated with increased alcohol use and alcohol problems among people who were vulnerable because they had positive alcohol expectancies or used avoidance coping^1^ (Cooper et al. 1988, 1990, 1992). Avoidance coping independently predicted drinking to cope and influenced the effect of stressors on this key alcohol measure. That is, people who scored high on avoidance coping also tended to score high on drinking to cope, and relationships between stressors and drinking to cope tended to be stronger for people who scored high on avoidance coping. Furthermore, alcohol expectancies influenced the predictive value of avoidance coping (i.e., avoidance coping was predictive of drinking to cope only among drinkers who held positive expectancies). A general tendency toward drinking to cope was a powerful predictor of both alcohol use and abuse. These findings strongly support the social learning model, a theory maintaining that personal factors, environment, and behavior are determinants of each other.

**Figure:**
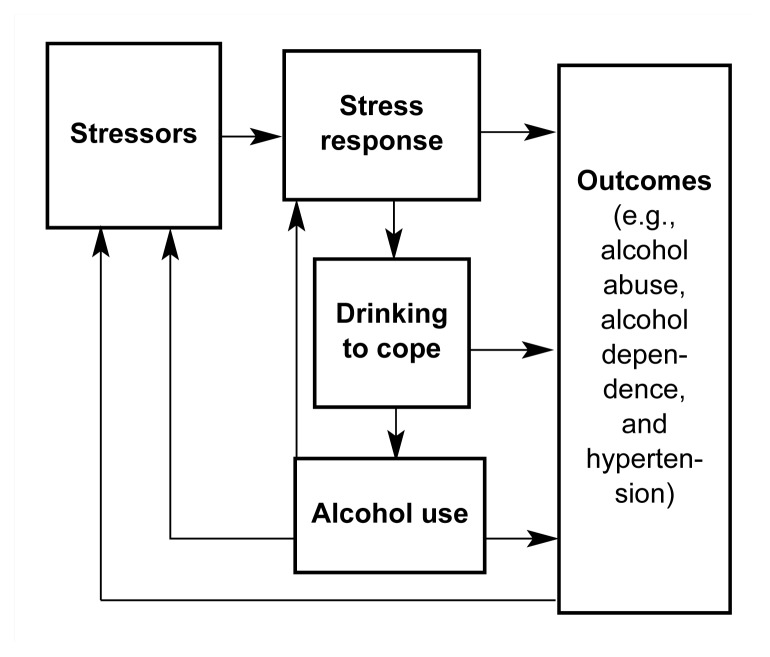
A model of the effects of stress on alcohol use and the effects of both stress and alcohol use on alcohol abuse, alcohol dependence, and hypertension.

**Table:** Number of Subjects Interviewed at Times 1, 2, and 3 by Race and Gender^1^

	Black		Non-Black		Total No. of Subjects
	Male	Female	Male	Female	
	
Time 1	373	686	359	515	1,933
Time 2	298	589	293	435	1,615
Time 3	261	525	256	385	1,427

1 The numbers shown are unweighted.

Peirce and colleagues (1996) further analyzed financial stress to examine the extent to which specific aspects of perceived social support influenced the relationship between financial stress and alcohol involvement. The surveys examined the following aspects of perceived social support: tangible social support (i.e., material aid), appraisal social support (i.e., the availability of a trusted advisor), and belonging social support (i.e., the availability of someone with whom the respondent could socialize or relax). Longitudinal regression analyses (see glossary, p. 305) conducted over two time periods supported the hypothesis that tangible social support, but not appraisal or belonging social support, reduced the effects of both acute and chronic financial stress on alcohol involvement.

#### Mediators of the Relationship Between Stress and Alcohol Use

As indicated in the model, stress responses, notably negative affect or depressive symptoms, were hypothesized to mediate the relationship between stressors and alcohol outcomes. Depressive symptoms completely explained the link between acute financial stressors and alcohol outcomes and partially explained the link between chronic financial stressors and alcohol outcomes (Peirce et al. 1994).

Although such social resources as perceived social support and social contacts appeared to moderate the relationship between stress and alcohol outcomes, research documenting a negative relationship between social resources and stress responses has suggested that deficits in social resources may themselves be stressors. According to longitudinal analyses, social resources are negatively related to depressive symptoms, which are, in turn, positively related to alcohol use (Peirce et al. in press). However, this model also suggested a feedback effect: Increases in alcohol use reduced subsequent contact with one’s social network. Additional longitudinal analyses revealed that depressive symptoms could predict alcohol problems, although the analysis did not provide evidence that alcohol problems predicted depressive symptoms over time (Moscato et al. 1997).

#### Gender and Racial Differences in the Relationship Between Stress and Alcohol Use

As indicated earlier, study results showed that vulnerability factors moderated the relationship between stressors and alcohol outcomes (Cooper et al. 1992). Gender also influenced this relationship. On average, negative life events, problems in the past month, and alcohol use were strongly related among men who scored high on both avoidance coping and positive alcohol expectancies. In contrast, negative life events and problems in the past month were not related to alcohol use among men who were low in both vulnerability factors or among women, regardless of their vulnerability status.

The results suggested a potentially important refinement of the widely accepted view that men as a group drink more heavily and experience more alcohol problems than do women. Increases in alcohol use related to stress were found only among men who reported using avoidance coping and/or having positive alcohol expectancies. When race was considered, the findings suggested that avoidance coping was a more potent vulnerability factor among blacks than among non-blacks. In contrast, strong positive alcohol expectancies proved to be a significant vulnerability factor for both blacks and non-blacks.

To investigate the impact of gender and race on the links proposed in their model, Peirce and colleagues (1994) conducted a cross-sectional analysis using the data collected at time 1. They examined the relationships between five factors and depressive symptoms: social contact, perceived social support, acute financial problems, chronic financial strain, and an index of personal resources constructed from measures of mastery^2^ and self-esteem. The researchers also examined four additional relationships—depression and drinking to cope, drinking to cope and the two alcohol outcomes (consumption and alcohol problems), and alcohol consumption and alcohol problems—for a total of nine. Of the nine relationships, six were significantly stronger among men in general than for women in general (without regard for race), and six were significantly stronger for blacks in general compared with non-blacks in general (without regard to gender). These findings suggested that depressive symptoms were influenced more by financial stressors and other factors among men compared with women and among blacks compared with non-blacks, and that depressive symptoms had a greater influence on alcohol consumption and alcohol problems among men than among women and among blacks than among non-blacks. In contrast, longitudinal analyses indicated that depressive symptoms at times 1 and 2 predicted alcohol problems at times 2 and 3, respectively, among women, but not among men (Moscato et al. 1997). The longitudinal analyses employed dichotomous variables that defined clinically significant levels of depressive symptoms and alcohol problems, whereas continuous measures of these variables were employed in the cross-sectional analyses. Possibly, subclinical levels of depressive symptoms play a greater role in men’s alcohol involvement than in women’s, but clinical levels of depressive symptoms may influence severe alcohol problems among women more than among men. Our longitudinal analyses, however, covered fairly lengthy time periods, and possibly, the influence of depressive symptoms on alcohol problems is more enduring among women, especially when high levels of both are concerned.

Frone and colleagues (1994) found that negative life events were positively related to the use of cigarettes, alcohol, and psychotherapeutic drugs but not to illicit drugs. No gender differences were found in the relationship between negative life events and cigarette use. However, negative life events were more strongly associated with alcohol use among men than among women but were more strongly associated with psychotherapeutic drug use among women than among men.

These findings raised the possibility that gender differences in alcohol consumption could be explained by gender differences in the relationship between stress and alcohol use. This possibility was ruled out by regression analyses (Cooper et al. 1997) indicating that when significant effects of gender were found, controlling for stress and coping factors increased rather than decreased the magnitude of gender differences in alcohol-related outcomes. Therefore, gender differences in stress and coping, at least as measured in the present data set, cannot be used to explain the fact that men drink more than women and experience more alcohol problems.

#### Summary of the Relationship Between Stress and Alcohol Use

Studies of the links between stressors and alcohol outcomes revealed that some people were more likely than others to cope with stress by drinking alcohol. Those people who drank in order to cope were likely to avoid confronting their problems and believed that drinking alcohol would have a positive effect. Men, including both blacks and non-blacks, appeared to be more vulnerable to these factors than women; blacks, including both men and women, appeared to be more vulnerable than non-blacks. (See the sidebar below for a discussion of some of these issues.)

**Motivation for Drinking**

Alcohol researchers have focused predominantly on the motivation to consume alcohol to cope with negative emotions rather than for social reasons or to enhance positive emotions. Cooper and colleagues (1992) found that each of the three drinking motives predicted a distinct and theoretically relevant set of outcomes. For example, enhancement motives most strongly predicted alcohol use, but coping motives most strongly predicted alcohol-related problems. The measure of alcohol-related problems was based on 17 symptoms of alcohol abuse or dependence grouped into three categories: (1) a pattern of pathological abuse (e.g., needing a drink before breakfast), (2) impairment of social or occupational functioning (e.g., experiencing trouble on the job or at school because of drinking), and (3) evidence of tolerance or withdrawal (e.g., experiencing the “shakes” after reducing or ceasing drinking). Although enhancement motives were related to both social and occupational impairment and pathological consumption, these relationships were mediated by alcohol use.

A subsequent analysis suggested that enhancement and coping are two distinct motives for alcohol use and that the effects of alcohol-related expectancies, emotions, and other individual differences on alcohol use are mediated through those two motives (Cooper et al. 1995). The effects of social motives were consistent across race and gender groups. In contrast, however, coping motives were more strongly related to alcohol outcomes among blacks, whereas enhancement motives were more strongly related to alcohol outcomes among non-blacks. The findings indicate that drinking to cope with negative emotions fits into a broader conceptualization of motives for alcohol use and their consequences (Cooper et al. 1992).

—Marcia Russell, M. Lynne Cooper, Michael R. Frone, and Robert S. Peirce

ReferencesCooperMLRussellMSkinnerJBWindleMDevelopment and validation of a three-dimensional measure of drinking motivesPsychological Assessment421231321992CooperMLFroneMRRussellMMudarPDrinking to regulate positive and negative emotions: A motivational model of alcohol useJournal of Personality and Social Psychology69599010051995747304310.1037//0022-3514.69.5.990

Some relationships between stressors and alcohol use were found to be mediated by depressive symptoms. Whereas a lack of social resources may have served as a stressor by increasing depressive symptoms, having social resources may have buffered the effect of a stressor, as in the case of tangible social support buffering the negative effects of financial stress. Thus, an understanding of drinking to cope and negative affect contributed to the understanding of stress-related alcohol use and alcohol problems among vulnerable people. Similar findings related to work stress and work-family conflict are discussed elsewhere in this issue (see the article in this issue by Frone, pp. 284–291).

This study also provided valuable information on the co-occurrence of alcohol disorders and depression. As predicted in the model, depressive symptoms mediated the link between financial stressors and drinking to cope, and drinking to cope mediated the relationship between depressive symptoms and alcohol use and related problems. Gender differences in the strength of the relationship between depressive symptoms and alcohol problems were influenced by the severity of the conditions and the length of the intervals involved, with the full range of the measures including subclinical conditions being more strongly related among men in cross-sectional analyses and clinically significant conditions being more strongly related among women in longitudinal analyses.

### Alcohol Use and Blood Pressure

This section examines the relationship between alcohol use and blood pressure, with an emphasis on the influence of different drinking patterns and racial differences on this relationship.

#### Drinking Patterns and Blood Pressure

Although research has established a positive relationship between the volume of alcohol consumed (e.g., drinks per week) and elevated blood pressure, the influence of drinking patterns has been relatively neglected (Puddey et al. 1999). The current study, however, examined the influence of drinking patterns on blood pressure (Russell et al. 1991) by considering the independent and interactive influences of quantity and frequency of alcohol intake on blood pressure. The study controlled for age, race, sex, body mass index (a measure of weight that considers height), marital status, education, salt and calcium intake, family medical history, medication to treat hypertension, smoking, and exercise. Frequency of alcohol consumption, compared with quantity, more strongly predicted both systolic blood pressure (SBP) and diastolic blood pressure (DBP).^3^ The mean SBP was 6.6 mmHg higher and the DBP was 4.7 mmHg higher among daily drinkers than among people who only drank once per week. Epidemiological studies have shown that mean elevations in BP of this magnitude are associated with significantly increased risk of stroke and coronary heart disease (Russell et al. 1991).

#### Racial Differences and the Relationship Between Alcohol Use and Blood Pressure

We used an approach developed by Malarcher and colleagues (1992) to investigate racial differences in the influence of usual and recent alcohol intake on blood pressure. Usual intake was defined as the average number of drinks per day in the 12 months preceding the blood pressure assessment. Recent intake was defined in terms of number of standard drinks^4^ consumed and time since the last drinking occasion among people who had consumed alcohol during the 48 hours before the blood pressure assessment. Both usual and recent alcohol use were positively and significantly related to SBP and DBP, and the increase in SBP associated with recent alcohol intake was greater among non-blacks than blacks. Race moderated the influence of acute alcohol intake on blood pressure: Among blacks who had had one or two drinks within the 48 hours before the interview, the average DBP and SBP values tended to decrease slightly with time since the last drink; but among blacks having three drinks or more, the mean values of DBP and SBP increased with time, peaking approximately 24 hours after the last drink, a pattern consistent with a mild withdrawal effect. The increase in blood pressure among blacks associated with having three or more drinks was stronger for SBP than for DBP.

A series of regression equations controlling for 12 covariates (see glossary, p. 305) was used to determine the influence of average drinks per day at time 1 on the SBP, DBP, and incidence of hypertension at time 3, taking race into consideration. Alcohol consumption at time 1 was weakly and positively associated with SBP at time 3 among both blacks and non-blacks, but it was only associated with higher DBP and risk of developing hypertension among non-blacks. In contrast, an increase in alcohol consumption between times 1 and 3 was associated with significant increases in SBP and risk of developing hypertension in both blacks and non-blacks. This finding is consistent with experimental and clinical studies showing that blood pressure changes fairly rapidly, perhaps within a few weeks, in response to changes in alcohol consumption.

#### Summary of the Relationship Between Alcohol Use and Blood Pressure

In an observational study, such as this, it is difficult to disentangle the effects of usual drinking pattern and recent drinking on blood pressure. Thus, people who drink frequently are more likely than infrequent drinkers to have been drinking during the 48 hours prior to being interviewed, and recent drinking accounts for some, but not all, of the variability in blood pressure associated with usual drinking patterns. However, to dismiss the observed elevations in blood pressure as an artifact of recent drinking is to miss the point that frequent drinking produces repeated elevations in blood pressure that over time damage the cardiovascular system in ways comparable to those caused by sustained hypertension. Also, although our analyses of usual drinking indicate that frequency of drinking accounted for more of the variability in blood pressure than did quantity, the data on recent drinking clearly indicate the importance of quantity, particularly among black respondents who consumed three or more drinks in the 48 hours before their interviews. Finally, our longitudinal analyses suggest that alcohol drinking has long-term effects on blood pressure and hypertension risk but that these effects may be partially reversible. These findings highlight the importance of abstaining or reducing the frequency and quantity of alcohol consumption to control high blood pressure.

#### Stress and Blood Pressure

Relatively little evidence for a direct impact of stressors or stress responses on blood pressure was found. However, Frone and colleagues (1997) found that among people employed at least 20 hours per week and with at least one child living at home, those who experienced stress as a result of family life interfering with work were more likely to have developed hypertension by the 4-year followup. The risk of developing hypertension was also predicted by parental stressors (e.g., parents feeling that their children made too many demands on them) and by two stress responses—anxiety and negative affect. These findings warrant further investigation as well as analyses to determine whether taking moderators into account will identify people who are vulnerable to the influence of stressors on blood pressure.

In addition, the epidemiologic literature on stress and cardiovascular disease implicates certain personality traits, such as anger and styles of coping with anger, in the etiology of hypertension. Given that anger is a personality trait rather than a stressor, it was conceptualized as a moderator in the model. However, the underlying implication of studies relating anger to blood pressure, (i.e., that high levels of trait anger and dysfunctional styles of coping with anger are proxy measures of stressful, anger-related responses to the hassles of daily living) seems plausible. Though not reviewed here, three additional studies have examined the extent to which alcohol consumption moderates or mediates the effects of anger on blood pressure and hypertension (Harburg et al. 1991; Russell et al. 1994*a*, 1995).

## Additional Studies

This study assessed many factors thought to influence stress, alcohol outcomes, and blood pressure. The three surveys generated a valuable data set that has been used to investigate many questions beyond the specific aims of the funded research. Those subjects include the following:

- Racial differences in factors associated with abstention or heavy drinking according to gender (Darrow et al. 1992; Mudar et al. 1992)
- Racial differences in correlates of alcohol problems (Russell et al. 1992)
- The influence of social networks on drinking (Bullers et al. 1995)
- The role of calcium in the relationship between alcohol use and blood pressure (Freudenheim et al. 1991)
- Alcohol’s involvement, if any, in a relationship between melatonin and blood pressure (Gleiberman et al. 1990, 1993)
- The influence of family history of alcoholism or problem drinking on alcohol disorders in offspring (Russell et al. 1990)
- The role of anger and dysfunctional anger coping styles in the etiology of alcoholism among children of parents who either were alcoholic or had drinking problems (Russell et al. 1993)
- The role of anger (Frone et al. 1993*a*) and marital anger (Russell et al. 1994*b*) in moderating the relationship between alcohol consumption and alcohol-related problems
- Methodological issues related to assessing alcohol expectancies (Frone et al. 1993*b*; George et al. 1995) and socially desirable responding (i.e., answering questions in a way that one thinks a listener would approve) (Welte and Russell 1993).

## Conclusions

The results of this study support a causal relationship between stress and alcohol use and demonstrate the importance of considering moderating and mediating factors when investigating the impact of life stressors on alcohol outcomes. Vulnerability factors, avoidance coping, and positive alcohol expectancies appear to play a major role in determining the impact of negative life events, problems, and financial stress on drinking to cope, increased alcohol use, and alcohol problems, especially among men. Depressive symptoms may mediate the relationship between some stressors and drinking to cope, and drinking to cope may mediate the relationship between depressive symptoms and both alcohol use and alcohol problems.

These findings have been influential in guiding research in this area and have practical implications for case finding and the development of effective treatment strategies. Avoidance coping and positive alcohol expectancies may be useful for identifying men at risk of developing alcohol problems in response to stressful life circumstances. The findings also suggest that cognitive restructuring interventions (i.e., interventions that address alcohol expectancies and skills training to develop more adaptive ways of coping with negative emotions) may be more effective than attempts to teach problem-focused coping skills, especially among men of all races and blacks of both genders.

Although women seemed less vulnerable to stress-induced alcohol outcomes, their risk of developing future alcohol problems increased when they experienced symptoms of depression, suggesting that treatment of depression in women may be effective in preventing alcohol problems.

The study found evidence to indicate that stressors and stress responses directly affected the incidence of hypertension, but average blood pressure did not appear to be similarly influenced, perhaps because the affected subjects were taking antihypertensive medication. The literature in this area, however, suggests other models that may be useful in extending the current investigation (Grim et al. 1995). For example, another possible model could examine the direct effects of trait anger and dysfunctional anger coping styles on drinking to cope, rather than the potential of these characteristics to moderate links between stress responses and drinking to cope. Similarly, further research can more fully investigate the extent to which deficits in social and personal resources may serve as stressors or moderate the effect of stressors on blood pressure.

The findings reported here confirm and extend previous reports that alcohol use contributes significantly to elevated blood pressure. Evidence that drinking patterns are important in determining the effect of alcohol consumption on blood pressure has important implications for the development of moderate drinking guidelines in the United States. Current guidelines are based on research that has not, for the most part, taken drinking patterns into account. The relationship between an episode of heavier drinking (i.e., consuming three or more drinks) and increased blood pressure among blacks is consistent with research indicating that a pattern of binge drinking may be associated with increased risk for heart disease. Further study is needed to characterize the health and psychosocial consequences associated with drinking patterns prevalent in the United States. This rich database continues to provide opportunities to investigate these important issues.

## Footnotes

1 Avoidance coping is an avoidant, emotion-focused coping style characterized by such behavior as taking negative feelings out on other people or trying to reduce tension by eating, smoking, or taking drugs.

2 Mastery assesses the extent to which people feel in control and able to cope with their lives.

3 Blood pressure is the force of blood against the walls of the arteries. Systolic pressure occurs during a heartbeat and is higher than diastolic pressure, which is the pressure in the arteries between heartbeats.

4 One standard drink was defined as 12 ounces (oz) of beer, 4 oz of wine, or 1 oz of distilled spirits.

## Glossary

Covariates: Factors that are associated with key study variables but are not of primary interest in a given analysis. Statistical adjustment for the influence of covariates on key study variables may be made to clarify relationships of primary interest.
Regression analysis: A statistical procedure used to determine the functional relationship between two or more correlated variables.
Regression equation: A formula derived from data to describe the relationship between two or more correlated variables, specifically to predict the average value of one variable when given the value(s) of the other variable(s).
